# Clinical assessment of T2 papillary thyroid carcinoma: a retrospective study conducted at a single tertiary institution

**DOI:** 10.1038/s41598-022-17979-2

**Published:** 2022-08-08

**Authors:** Hyesung Kim, Kwangsoon Kim, Ja Seong Bae, Jeong Soo Kim

**Affiliations:** Department of Surgery, College of Medicine, The Catholic University of Korea, Seoul, 06591 Republic of Korea

**Keywords:** Cancer, Endocrinology, Oncology

## Abstract

The extent of surgery among patients with T2 papillary thyroid carcinoma (PTC) remains controversial. Thus, we herein aimed to evaluate the risk factors for recurrence, particularly based on the extent of surgery, among patients with T2 PTC at a single tertiary institution. We assessed 251 patients who underwent thyroid surgery for T2 PTC from January 2009 to December 2014 at Seoul St. Mary’s Hospital (Seoul, Korea). The mean follow-up duration was 100.7 months. Eleven (4.4%) patients had recurrence. The recurrence rates did not significantly differ in terms of the extent of surgery (p = 0.868). Patients with a high lymph node ratio (LNR) had a significantly higher recurrence rate than those with a low LNR (p < 0.001). According to a recurrence pattern analysis, five of six patients in the lobectomy group had recurrence in the ipsilateral lateral compartment. A multivariate analysis revealed that a high LNR was a significant risk factor for recurrence (hazard ratio: 11.025, p = 0.002). Our results suggest that patients without clinical evidence of any lymph node metastases and those with limited lesions in the thyroid gland can undergo lobectomy and LNR can serve as an independent risk factor for predicting recurrence in T2 PTC.

## Introduction

Thyroid cancer is the most common endocrine malignancy, and its incidence has been significantly increasing worldwide in the last several decades^1–4^. Papillary thyroid carcinoma (PTC) is the most frequent malignancy of the thyroid gland, which accounts for 80%–90% of all thyroid malignancies. Moreover, follicular thyroid carcinoma is the second most common malignancy^5^. According to cancer statistics, recently, thyroid cancer has been the most frequently diagnosed in Korea^6^. PTC has an excellent prognosis due to its indolent features, and the overall survival rate is > 90%^7^.

Generally, PTC is treated with surgery and postoperative management, including thyroid-stimulation hormone suppression and/or radioactive iodine (RAI) ablation. Surgery is the main treatment for PTC. The extent of surgery ranges from lobectomy to total thyroidectomy (TT), and it has been a controversial topic for a long time. Barney et al. showed that the efficacy of lobectomy and TT did not differ^8^. The National Cancer Database showed that the prognosis of patients with PTC measuring 1–4 cm who underwent lobectomy did not significantly differ from that of patients who underwent TT^9^. The American Thyroid Association (ATA) management guidelines recommend lobectomy for patients with PTC measuring 1–4 cm without extrathyroidal extension (ETE) and clinical evidence of any lymph node (LN) metastasis^10^.

If the recurrence and survival rates of lobectomy are similar to those of TT, it can be used for treatment. The disadvantages of TT may lead to different complications. Transient/persistent postoperative hypoparathyroidism and recurrent laryngeal nerve injury are more frequent and severe after TT than after lobectomy^11,12^. To date, the extent of surgery among patients with T2 PTC remains controversial. Thus, an accurate preoperative risk evaluation is important to determine the extent of surgery.

To the best of our knowledge, only few studies have reported the clinical characteristics and prognosis of T2 PTC. Hence, this current retrospective study aimed to evaluate the risk factors for recurrence, particularly based on the extent of surgery, in patients with T2 PTC at a single tertiary institution.

## Results

### Comparison of baseline clinicopathological characteristics between the TT and lobectomy groups

Table 1 shows the baseline clinicopathological characteristics of the TT and lobectomy groups. The mean tumor size did not significantly differ between the two groups (2.7 ± 0.5 vs. 2.8 ± 0.6 cm, p = 0.197). Further, there were no statistically significant differences in terms of sex, multifocality, vascular and perineural invasion, BRAF positivity, and TNM stages. However, the lobectomy group was significantly older than the TT group (p = 0.017). In contrast, the proportion of patients with bilaterality and minimal ETE, which is an extension to the thyroid capsule, perithyroidal soft tissue, or sternothyroid muscle^13^, was significantly higher in the TT group than that in the lobectomy group (p < 0.001 and p = 0.003, respectively). The TT group had a significantly higher N stage than the lobectomy group (p < 0.001). Five (4.6%) patients in the TT group and 6 (4.2%) in the lobectomy group had recurrence. However, the recurrence rates did not significantly differ (p = 0.868).

**Table 1:** Baseline clinicopathological characteristics according to the extent of surgery.

	Lobectomy (n = 143)	TT and/or mRND (n = 108)	p-value
Age (years)	44.8 ± 15.0	40.4 ± 13.1	0.017
Male sex	41 (28.7%)	39 (36.1%)	0.221
Tumor size (cm)	2.7 ± 0.5	2.8 ± 0.6	0.197
Minimal ETE	54 (37.8%)	61 (56.5%)	0.003
Multifocality	49 (34.3%)	48 (44.4%)	0.117
Bilaterality	0 (0%)	40 (37.0%)	< 0.001
Lymphatic invasion	46 (32.2%)	61 (56.5%)	< 0.001
Vascular invasion	8 (5.6%)	9 (8.3%)	0.451
Perineural invasion	4 (2.8%)	1 (0.9%)	0.394
BRAF positivity	80/115 (69.6%)	61/78 (15.2%)	0.247
Harvested LNs	10.0 ± 7.3	27.5 ± 26.4	< 0.001
Positive LNs	2.3 ± 3.6	7.2 ± 7.8	< 0.001
**N stage**			< 0.001
N0	76 (53.1%)	28 (25.9%)	
N1a	67 (46.9%)	46 (42.6%)	
N1b	0 (0%)	34 (31.5%)	
**TNM stage**			0.887
I	129 (90.2%)	98 (90.7%)	
II	14 (9.8%)	10 (9.3%)	
Recurrence	6 (4.2%)	5 (4.6%)	0.868

Data were expressed as number (%) or mean ± standard deviation.

A statistically significant difference was defined as p < 0.05.

*TT* total thyroidectomy, *mRND* modified radical neck dissection, *ETE* extrathyroidal extension, *LN* lymph node, *T* tumor, *N* node.

### Comparison of baseline clinicopathological characteristics according to sex, tumor size, age, and LNR

Table 2 shows the baseline clinicopathological characteristics according to sex. There was no statistically significant difference in terms of the extent of surgery between the two groups. The female group had a higher multifocality and bilaterality than the male group (p = 0.001 and p = 0.005, respectively). Meanwhile, the proportion of patients with positive LNs was significantly higher in the male group than in the female group (p = 0.006). However, there was no significant difference in terms of recurrence rates between the two groups (5.0% vs. 4.1%, p = 0.748).

**Table 2:** Baseline clinicopathological characteristics according to sex.

	Male (n = 80)	Female (n = 171)	p-value
Age (years)	44.5 ± 13.9	42.2 ± 14.5	0.229
**Extent of surgery**			0.221
Lobectomy	41 (51.3%)	102 (59.6%)	
TT and/or mRND	39 (48.7%)	69 (40.4%)	
Tumor size (cm)	2.8 ± 0.6	2.7 ± 0.5	0.089
Minimal ETE	39 (48.7%)	76 (44.4%)	0.587
Multifocality	19 (23.8%)	78 (45.6%)	0.001
Bilaterality	5 (6.3%)	35 (20.5%)	0.005
Lymphatic invasion	35 (43.8%)	72 (42.1%)	0.891
Vascular invasion	8 (10.0%)	9 (5.3%)	0.183
Perineural invasion	0 (0%)	5 (2.9%)	0.181
BRAF positivity	42/57 (73.7%)	99/136 (72.8%)	0.899
Harvested LNs	20.5 ± 20.7	16.2 ± 19.7	0.117
Positive LNs	5.9 ± 6.7	3.6 ± 5.9	0.006
**N stage**			0.340
N0	29 (36.3%)	75 (43.9%)	
N1a	37 (46.2%)	76 (44.4%)	
N1b	14 (17.5%)	20 (11.7%)	
**TNM stage**			0.645
I	71 (88.8%)	156 (91.2%)	
II	9 (11.2%)	15 (8.8%)	
Recurrence	4 (5.0%)	7 (4.1%)	0.748

Data were expressed as number (%) or mean ± standard deviation.

A statistically significant difference was defined as p < 0.05.

*TT* total thyroidectomy, *mRND* modified radical neck dissection, *ETE* extrathyroidal extension, *LN* lymph node, *T* tumor, *N* node.

As shown in Table 3, patients were divided into two groups according to tumor size: small tumor group (≤ 3 cm, n = 190 [75.7%]) and large tumor group (> 3 cm, n = 61 [24.3%]). Nevertheless, the two groups did not significantly differ in terms of age, extent of surgery, bilaterality, lymphatic invasion, perineural invasion, BRAF positivity, number of harvested LNs and positive LNs, and TNM stage. The large tumor group had a higher proportion of male patients than the small tumor group (28.4% vs. 42.6%, p = 0.042). The large tumor group had a significantly higher N stage than the small tumor group (p = 0.039). In addition, vascular invasion was more common in the large tumor group than in the small tumor group (4.7% vs. 13.1%, p = 0.037). However, there was no significant difference in terms of recurrence rate between the two groups (3.2% vs. 8.2%, p = 0.142).

**Table 3:** Baseline clinicopathological characteristics according to tumor size.

	Tumor size ≤ 3 cm (n = 190)	Tumor size > 3 cm (n = 61)	p-value
Age (years)	43.0 ± 14.2	42.6 ± 14.8	0.842
Male sex	54 (28.4%)	26 (42.6%)	0.042
**Extent of surgery**			0.459
Lobectomy	111 (58.4%)	32 (52.5%)	
TT and/or mRND	79 (41.6%)	29 (47.5%)	
Tumor size (cm)	2.5 ± 0.3	3.6 ± 0.3	< 0.001
Minimal ETE	96 (50.5%)	19 (31.1%)	0.012
Multifocality	81 (42.6%)	16 (26.2%)	0.024
Bilaterality	31 (16.3%)	9 (14.8%)	0.843
Lymphatic invasion	81 (42.6%)	26 (42.6%)	1.000
Vascular invasion	9 (4.7%)	8 (13.1%)	0.037
Perineural invasion	5 (2.6%)	0 (0%)	0.340
BRAF positivity	103/144 (71.5%)	38/49 (77.6%)	0.461
Harvested LNs	16.7 ± 17.6	20.3 ± 26.5	0.221
Positive LNs	4.1 ± 5.5	5.3 ± 8.3	0.214
**N stage**			0.039
N0	73 (38.4%)	31 (50.8%)	
N1a	94 (49.5%)	19 (31.1%)	
N1b	23 (12.1%)	11 (18.0%)	
**TNM stage**			0.933
I	172 (90.5%)	55 (90.2%)	
II	18 (9.5%)	6 (9.8%)	
Recurrence	6 (3.2%)	5 (8.2%)	0.142

Data were expressed as number (%) or mean ± standard deviation.

A statistically significant difference was defined as p < 0.05.

*TT* total thyroidectomy, *mRND* modified radical neck dissection, *ETE* extrathyroidal extension, *LN* lymph node, *T* tumor, *N* node, *M* metastasis.

Table 4 depicts the baseline clinicopathological characteristics according to age. The following groups were divided according to age: younger group (< 55 years, n = 199 [79.3%]) and older group (≥ 55 years, n = 52 [20.7%]). The younger group had a significantly higher proportion of patients with lymphatic invasion, BRAF positivity, and a greater number of harvested LNs and positive LNs than the older group (p = 0.004, p = 0.031, p = 0.042, and p = 0.021, respectively). The younger group had a significantly higher N stage than the older group (p = 0.009). However, the recurrence rates between the two groups did not significantly differ (5.0% vs. 1.9%, p = 0.468).

**Table 4:** Baseline clinicopathological characteristics according to age.

	Age < 55 years (n = 199)	Age ≥ 55 years (n = 52)	p-value
Age (years)	37.6 ± 10.5	63.1 ± 7.1	< 0.001
Male sex	65 (32.7%)	15 (28.8%)	0.738
**Extent of surgery**			0.208
Lobectomy	109 (54.8%)	34 (65.4%)	
TT and/or mRND	90 (45.2%)	18 (34.6%)	
Tumor size (cm)	2.7 ± 0.5	2.8 ± 0.6	0.826
Minimal ETE	93 (46.7%)	22 (42.3%)	0.640
Multifocality	71 (35.7%)	26 (50.0%)	0.078
Bilaterality	32 (16.1%)	8 (15.4%)	0.903
Lymphatic invasion	94 (47.2%)	13 (25.0%)	0.004
Vascular invasion	15 (7.5%)	2 (3.8%)	0.537
Perineural invasion	4 (2.0%)	1 (1.9%)	0.968
BRAF positivity	116/151 (76.8%)	25/42 (59.5%)	0.031
Harvested LNs	18.9 ± 20.5	12.5 ± 17.7	0.042
Positive LNs	4.8 ± 6.1	2.6 ± 6.6	0.021
**N stage**			0.009
N0	76 (38.2%)	28 (53.8%)	
N1a	91 (45.7%)	22 (42.3%)	
N1b	32 (16.1%)	2 (3.8%)	
**TNM stage**			< 0.001
I	199 (100%)	28 (53.8%)	
II	0 (0%)	24 (46.2%)	
Recurrence	10 (5.0%)	1 (1.9%)	0.468

Data were expressed as number (%) or mean ± standard deviation.

A statistically significant difference was defined as p < 0.05.

*TT* total thyroidectomy, *mRND* modified radical neck dissection, *ETE* extrathyroidal extension, *LN* lymph node, *T* tumor, *N* node, *M* metastasis.

Table 5 shows the baseline clinicopathological characteristics according to LNR. The optimal cutoff for LNR (0.32) was obtained via a receiver operating characteristic (ROC) curve analysis (Supplementary Fig. 1). The patients were classified into two groups: low LNR group (≤ 0.32, n = 176 [70.1%]) and high LNR group (> 0.32, n = 75 [29.9%]). There were no statistically significant differences in terms of extent of surgery, tumor size, bilaterality, vascular and perineural invasion, BRAF positivity, and number of harvested LNs. The high LNR group was significantly younger than the low LNR group (p = 0.006). The proportion of male patients and those with minimal ETE, lymphatic invasion, and positive LNs was higher in the high LNR group than in the low LNR group (p = 0.005, p = 0.001, p < 0.001, and p < 0.001, respectively). The high LNR group had a higher N and TNM stage than the low LNR group (p < 0.001 and p = 0.033, respectively). Moreover, the recurrence rate was significantly higher in the high LNR group than in the low LNR group (1.1% vs. 12.0%, p < 0.001).

**Table 5:** Baseline clinicopathological characteristics according to lymph node ratio.

	LNR ≤ 0.32 (n = 176)	LNR > 0.32 (n = 75)	p-value
Age (years)	44.5 ± 13.9	39.1 ± 14.7	0.006
Male sex	46 (26.1%)	34 (45.3%)	0.005
**Extent of surgery**			0.126
Lobectomy	106 (60.2%)	37 (49.3%)	
TT and/or mRND	70 (39.8%)	38 (50.7%)	
Tumor size (cm)	2.7 ± 0.5	2.8 ± 0.6	0.804
Minimal ETE	68 (38.6%)	47 (62.7%)	0.001
Multifocality	79 (44.9%)	18 (24.0%)	0.002
Bilaterality	32 (18.2%)	8 (10.7%)	0.187
Lymphatic invasion	43 (24.4%)	64 (85.3%)	< 0.001
Vascular invasion	11 (6.3%)	6 (8.0%)	0.593
Perineural invasion	3 (1.7%)	2 (2.7%)	0.637
BRAF positivity	96/136 (70.6%)	45/57 (53.3%)	0.287
Harvested LNs	17.3 ± 20.8	18.2 ± 18.4	0.734
Positive LNs	2.1 ± 4.4	9.6 ± 6.9	< 0.001
**N stage**			< 0.001
N0	104 (59.1%)	0 (0%)	
N1a	50 (28.4%)	63 (84.0%)	
N1b	22 (12.5%)	12 (16.0%)	
**TNM stage**			0.033
I	164 (93.2%)	63 (84.0%)	
II	12 (6.8%)	12 (16.0%)	
Recurrence	2 (1.1%)	9 (12.0%)	< 0.001

Data were expressed as number (%) or mean ± standard deviation.

A statistically significant difference was defined as p < 0.05.

*LNR* lymph node ratio, *TT* total thyroidectomy, *mRND* modified radical neck dissection, *ETE* extrathyroidal extension, *LN* lymph node, *T* tumor, *N* node, *M* metastasis.

### Recurrence patterns in the study population

Table 6 shows the recurrence patterns in the study population. Five patients in the lobectomy group had recurrence at the ipsilateral lateral compartment. Only one patient developed recurrence at the contralateral thyroid gland. By contrast, patients in the TT group presented with different recurrence patterns, which are as follows: patient no. 1 at the bilateral lateral compartment, patient no. 2 at the ipsilateral lateral compartment, patient no. 3 at the ipsilateral OP bed, patient no. 4 at the ipsilateral central compartment, and patient no. 5 at the contralateral lateral compartment.

**Table 6:** Recurrence patterns of the study population.

Patients	Age	Sex	Tumor size (cm)	Recurrence site	DFS (months)
**TT**					
1	21	Female	3.2	Bilateral level 4 LNs	7
2	23	Female	2.3	Ipsilateral level 4 LNs	6
3	33	Female	4	Ipsilateral op bed	100
4	37	Male	3.7	Ipsilateral level 6 LNs	40
5	32	Male	4	Contralateral level 4 LNs	39
**Lobectomy**					
1	38	Female	2.5	Contralateral thyroid	38
2	47	Female	2.5	Ipsilateral level 3 LNs	8
3	24	Female	3	Ipsilateral level 4 LNs	12
4	23	Female	2.3	Ipsilateral level 4 LNs	6
5	36	Male	3.5	Ipsilateral level 3 LNs	23
6	62	Male	2.3	Ipsilateral level 4 LNs	7

All patients who underwent BTT received radioactive iodine ablation therapy.

*DFS* disease-free survival, *TT* total thyroidectomy, *LN* lymph node.

### Univariate and multivariate analyses of the risk factors for recurrence

Table 7 shows the results of the univariate and multivariate Cox regression analyses of the risk factors for disease-free survival (DFS). Age (hazard ratio [HR] 0.951; p = 0.041), minimal ETE (HR 5.347; p = 0.032), lymphatic invasion (HR 6.256; p = 0.019), positive LNs (HR 1.087; p = 0.001), and high LNR (HR 17.168, p = 0.006) were found to be a significant predictor of recurrence in the univariate analysis. Based on the multivariate analysis, only high LNR was a significant risk factor for recurrence (HR 8.603, p = 0.007).

**Table 7:** Univariate and multivariate analyses of disease-free survival.

	Univariate		Multivariate	
	HR (95% CI)	p-value	HR (95% CI)	p-value
Age	0.951 (0.907–0.998)	0.041		
Minimal ETE	5.347 (1.154–24.778)	0.032		
Lymphatic invasion	6.256 (1.351–28.954)	0.019		
Positive LNs	1.087 (1.034–1.143)	0.001		
**LNR**	17.168 (2.245–131.307)	0.006		
≤ 0.32	Ref.		Ref.	
> 0.32	11.025 (2.381–51.039)	0.002	8.603 (1.822–40.628)	0.007

Data were expressed as hazard ratio (HR) and 95% confidence interval (CI).

A statistically significant difference was defined as p < 0.05.

*ETE* extrathyroidal extension, *LN* lymph node, *LNR* lymph node ratio.

**Figure 1:**
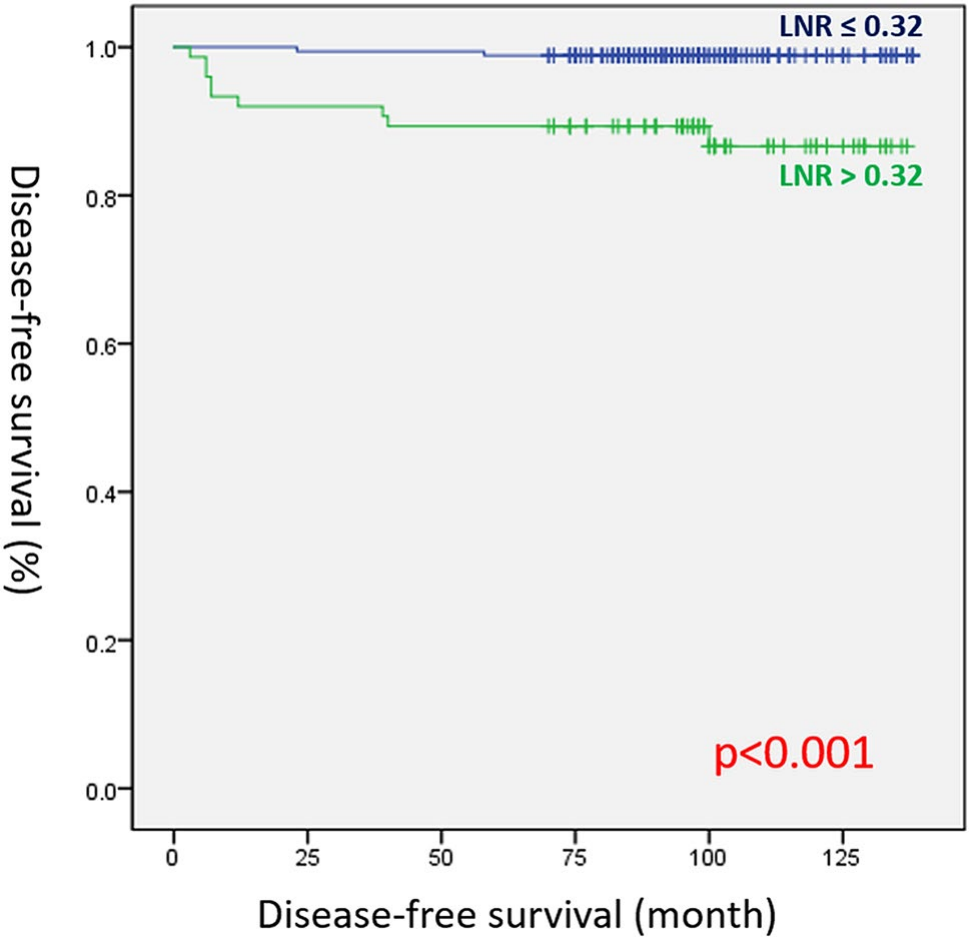
Disease-free survival curves according to lymph node ratio (LNR) (log-rank test, p < 0.001).

**Figure 2:**
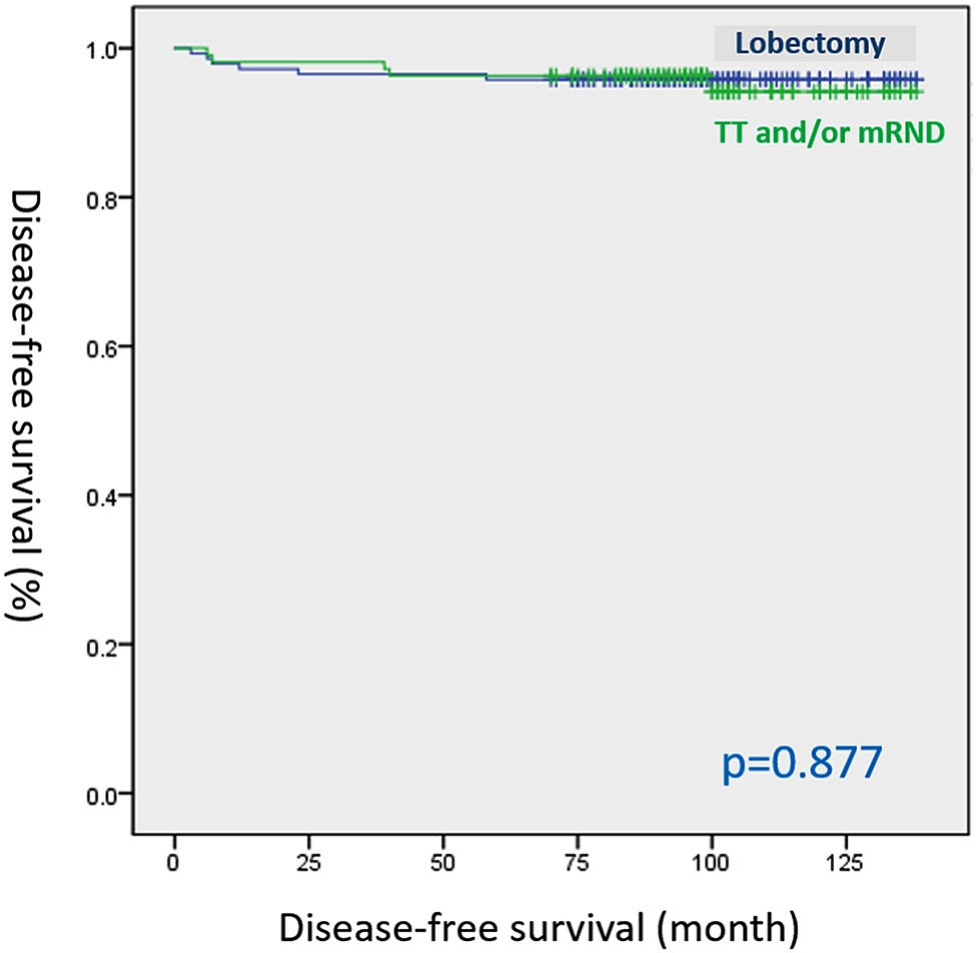
Disease-free survival curves according to the extent of surgery (log-rank test, p = 0.877). *TT* total thyroidectomy, *mRND* modified radical neck dissection.

In the Kaplan–Meier analysis, DFS differed significantly between the high and low LNR groups (log-rank test, p < 0.001; Fig. 1). However, there was no statistically significant difference in terms of DFS between the TT and lobectomy groups (log-rank test, p = 0.877; Fig. 2).

## Discussion

The American Joint Committee on Cancer/Union for International Cancer Control TNM staging system for thyroid cancer defines T category as follows: T0, no evidence of primary tumor; T1, size of ≤ 2 cm and intrathyroidal; T2, 2 cm < size ≤ 4 cm and intrathyroidal; T3, size of > 4 cm or ETE (sternohyoid, sternothyroid, thyrohyoid, and omohyoid muscle); T4, others–gross ETE. T2 accounts for only 3%–13% of all PTC cases in Korea^14–16^. The incidence of T1 PTC has increased due to improvements in diagnostic modalities and early screening in Korea^17^. As gross ETE is often observed in patients with thyroid cancer, T3 or T4 PTC accounts for a significant proportion^18^. Meanwhile, T2 PTC accounts for a relatively small portion of all PTC cases.

According to the ATA management guidelines, lobectomy alone may be sufficient when used as an initial treatment for patients with PTC measuring 1–4 cm but without ETE and clinical evidence of any LN metastases^10^. However, the extent of surgery for PTC measuring 1–4 cm is still controversial. After the publication of the 2015 ATA management guidelines, several studies investigated the extent of surgery in PTC measuring 1–4 cm^19–21^. Rajjoub et al. showed that lobectomy is not sufficient for T2 PTC. Results showed that 33,816 adults with conventional PTC measuring 1.0–3.9 cm had a better survival after TT than after lobectomy. This finding was observed particularly in patients with a tumor size of 2.0–3.9 cm^19^. Suman et al. revealed that lobectomy had a significantly negative effect on long-term survival. The exclusion of high-risk features is important when adopting lobectomy as the definitive surgical therapy for T1b and T2 PTC because of its potential adverse effects on long-term survival^22^. By contrast, previous research revealed that lobectomy might be appropriate for patients with low-risk differentiated thyroid carcinoma (DTC). Cautious risk evaluation and stratification can individualize treatment, prevent overtreatment, and guarantee a good long-term prognosis with a low-risk of recurrence^23^. Filippo et al. revealed no significant difference in terms of the risk of locoregional recurrence or distant metastasis between the TT and lobectomy groups who presented with pT1-T2 and pN0 PTC. Furthermore, compared with lobectomy, TT was correlated with more complications, which included postoperative hypoparathyroidism and recurrent laryngeal nerve injury^20^. Consistent with the study of Filippo, there was no statistically significant difference in terms of recurrence rate between the TT and lobectomy groups in our study (p = 0.868). The Kaplan–Meier analysis of DFS showed no significant difference between the two groups (log-rank test, p = 0.877).

If recurrence occurs commonly in the remnant thyroid gland, TT, rather than lobectomy, might be recommended. We analyzed the recurrence patterns in the study population. In the current study, five (4.6%) patients in the TT group and 6 (4.2%) in the lobectomy group were diagnosed with recurrence. All but one patient in the lobectomy group had recurrence at the ipsilateral lateral compartment. On the contrary, the recurrence pattern of the TT group varied. Thus, recurrence occurred mainly in the lateral compartment rather than the remnant thyroid gland after lobectomy.

TT is advantageous as it can improve surveillance accuracy using serum thyroglobulin as a sensitive postoperative marker for residual or recurrent thyroid cancer^24^. Moreover, it allows the use of RAI, which can be used in both postoperative treatment and surveillance^25^. RAI increases the survival rates of patients with intermittent- and high-risk DTC. However, the ATA management guidelines do not recommend RAI ablation in patients with low-risk T2 PTC^10^. Schvartz et al. showed that RAI after surgery has no survival benefit in a large cohort of patients with low-risk DTC^26^.

The extent of surgery should not be based on the risk of recurrence alone. TT can cause various postoperative complications. However, such complications are rare. First, TT is associated with a higher risk of hypoparathyroidism. After thyroidectomy, 19%–38% and 0%–3% of patients presented with transient and permanent hypoparathyroidism, respectively^27^. Permanent hypoparathyroidism is associated with multiple complications, including renal function impairment, gastrointestinal and neuropsychiatric problems, and infections^28^. Second, TT is also associated with a greater risk of recurrent laryngeal nerve injury. Approximately 0.5%–5% and 1%–30% of patients who undergo TT had permanent recurrent laryngeal nerve injury and temporary injury, respectively^29^. Third, patients with PTC are relatively young, mostly in their 40 s or 50 s at the time of diagnosis^30^. Considering that PTC has a good prognosis, patients must be treated with levothyroxine for about 30–50 years after TT. The long-term use of this drug causes complications including osteoporosis and arrhythmias^31,32^. Further studies about the complications of TT, which were not included in the current study, should be performed.

Since the purpose of this study was to observe the general characteristics of patients with T2 PTC, data on patients’ complications were not initially included. Among the patients included in this study, transient vocal cord palsy was observed in three patients (2.1%) in the lobectomy group and four patients (3.7%) in the TT group. There was no statistically significant difference in terms of transient vocal cord palsy between the two groups. Permanent vocal cord palsy was not observed in any patient in the lobectomy group, whereas it was observed in one (0.9%) patient in the TT group. Transient hypoparathyroidism was not found in the lobectomy group, whereas it occurred in 25 patients (23.1%) in the TT group. These findings are consistent with those reported in a recent meta-analysis^33^.

LNR is calculated by dividing the number of positive LNs by the number of harvested LNs, and it is used in predicting recurrence in other types of cancers^34,35^. Recently, LNR was found to be an important predictor of DFS in PTC^36^. Schneider et al. assessed 10,955 cases, and results showed that LNR was a strong prognostic factor^37^. Vas Nunes et al. conducted a retrospective analysis of 198 patients with PTC who underwent TT. Results showed that LNR was an important independent prognostic factor in PTC, and it could be used in combination with existing staging systems^38^. Our study found similar results. An ROC curve analysis was performed to obtain an optimal cutoff value of 0.32. A multivariate analysis found that an LNR of > 0.32 was a significant risk factor for recurrence. In the Kaplan–Meier analysis, the DFS between the high and low LNR groups did not significantly differ (log-rank test, p < 0.001). However, the optimal cutoff of LNR for the risk of recurrence in PTC is still controversial. Schneider et al. showed that a cutoff value of 0.42 can be used for risk stratification in patients with positive LNs^37^. Vas Nunes et al. proposed that an LNR cutoff value of 0.3 can be a prognostic factor^38^. In this study, the optimal cutoff value of LNR was 0.32. Thus, further prospective or multicenter studies must be conducted to determine the optimal cutoff value of LNR. The high LNR group was younger and had a higher number of male patients than the low LNR group. This result was consistent with that of several studies. Wang et al. showed that younger patients with a high LNR are at a greater risk for PTC^39^. Kim et al. performed a large cohort study. Results showed that male patients had a greater number of positive LNs^40^. Nevertheless, further studies should be conducted to determine the relevance of age and sex to LNR. This study identified the recurrence patterns of patients with T2 PTC. Most recurrences did not occur in the remnant thyroid gland after lobectomy. Even though postoperative pathologic results showed that patients had a high LNR, we do not routinely recommend thyroidectomy after lobectomy. Short-term follow-up may be helpful for patients with T2 PTC who have a high LNR after lobectomy.

The current study had several limitations due to its retrospective nature. First, the strength of the result was undermined. Second, the participants were from a single tertiary institution. Hence, this might have caused selection bias, and these participants might not reflect the entire patient population. Finally, the follow-up period was relatively short (100.7 ± 18.3 months). Hence, a longer follow-up is required to predict the long-term surgical outcomes of patients with T2 PTC, as it has indolent features. Nevertheless, these limitations could be addressed by conducting a multicenter study in the future.

However, the study also had some advantages. That is, each patient was followed-up, and standardized laboratory and imaging protocols from a single institution were used. To the best of our knowledge, only few studies have analyzed T2 PTC individually. Although other studies have already addressed PTC recurrence, this research differs as it has identified recurrence patterns in the TT and lobectomy groups. This then contributes to determining the extent of surgery.

In conclusion, lobectomy is not associated with a higher risk of recurrence and is feasible among patients with T2 PTC. Moreover, it may be considered for patients without ETE, suspicious LN metastasis, and intrathyroidal lesion. LNR can be an independent risk factor for recurrence in T2 PTC. Thus, short-term follow-up may be recommended for patients with T2 PTC who have a high LNR.

## Methods

### Patients

The data of 279 patients with T2 PTC who underwent thyroidectomy at Seoul St. Mary’s Hospital (Seoul, Korea) between January 2009 and December 2014 were retrospectively reviewed. In total, 10 and 18 patients were excluded from the analysis because of insufficient data and loss to follow-up, respectively. The data of 251 patients were completely analyzed by reviewing the medical charts and pathology reports. Among them, 108 (43.0%) underwent TT and/or modified radical neck dissection and 143 (57.0%), lobectomy. Prophylactic central LN dissection was routinely performed in all patients included in this study. The mean follow-up duration was 100.7 ± 18.3 (range 70–139) months. The study was conducted in accordance with the Declaration of Helsinki (as revised in 2013). Moreover, it was approved by the institutional review board of Seoul St. Mary’s Hospital, The Catholic University of Korea (IRB No.: KC21RISI0234). The need for informed consent was waived due to the retrospective nature of this research by the institutional review board of Seoul St. Mary’s Hospital, The Catholic University of Korea.

### Postoperative management and follow-up

All patients with T2 PTC were managed after surgical treatment according to the ATA management guidelines^10^. Patients were treated with levothyroxine at suppressive doses and were regularly followed-up. In addition, all patients underwent physical examination, thyroid function test, thyroglobulin (Tg), anti-Tg antibody concentration assessment, and neck ultrasonography every 3–6 months, and annually thereafter. Postoperative RAI ablation was performed at 6–8 weeks after surgery, and whole-body scans were performed at 5–7 days after RAI ablation in patients who underwent TT. To determine the location and extent of suspected recurrence, patients who had evidence of recurrence on routine follow-up evaluations were assessed via additional diagnostic imaging techniques, including computed tomography (CT), positron emission tomography/computed tomography, and/or RAI whole-body scanning. During the follow-up, patients were considered to have recurrent disease if either or both of the following were observed: (1) positive imaging findings on ultrasound-guided needle aspiration biopsy, CT, or diagnostic ^131^I whole-body scan and (2) significant increases (i.e. ≥ 50%) in the stimulated and/or basal serum Tg levels with respect to the previous visit(s)^41^. Recurrence was confirmed via histologic examination using ultrasound-guided needle aspiration biopsy or surgical biopsy.

### Statistical analysis

Continuous variables were presented as mean with standard deviation, and categorical variables as number with percentage. The student’s *t*-test was used to compare continuous variables. Meanwhile, categorical variables were compared using the Pearson’s chi-square test or the Fisher’s exact test. To determine the optimal cutoff value of the lymph node ratio (LNR), which is defined as the number of positive LNs divided by the total number of LNs harvested, ROC curve analysis was performed. Univariate and multivariate Cox regression analyses were performed to identify the predictors of DFS, which is defined as the length of time for which the patient survives without any signs or symptoms of the cancer after the completion of the primary treatment for the cancer, using calculated HRs with 95% confidence intervals. Kaplan–Meier survival analysis with log-rank test was performed to compare DFS. A p-value of < 0.05 was considered statistically significant. All statistical analyses were performed using the Statistical Package for the Social Sciences software for Windows version 23.0 (IBM Corp., Armonk, NY, USA).

## Supplementary Information

Supplementary Figure 1.

## Data Availability

The data that support the findings of this study are available on request from the corresponding author. The data are not publicly available due to privacy or ethical restrictions.
