# Supplementary material for: Clinical assessment of T2 papillary thyroid carcinoma: a retrospective study conducted at a single tertiary institution
Source: Sci Rep. 2022 Aug 8;12:13548. doi: 10.1038/s41598-022-17979-2 (PMC9360027; doi:10.1038/s41598-022-17979-2)
Supplement: Supplementary file 1 — Supplementary Figure 1. [file 41598_2022_17979_MOESM1_ESM.docx]

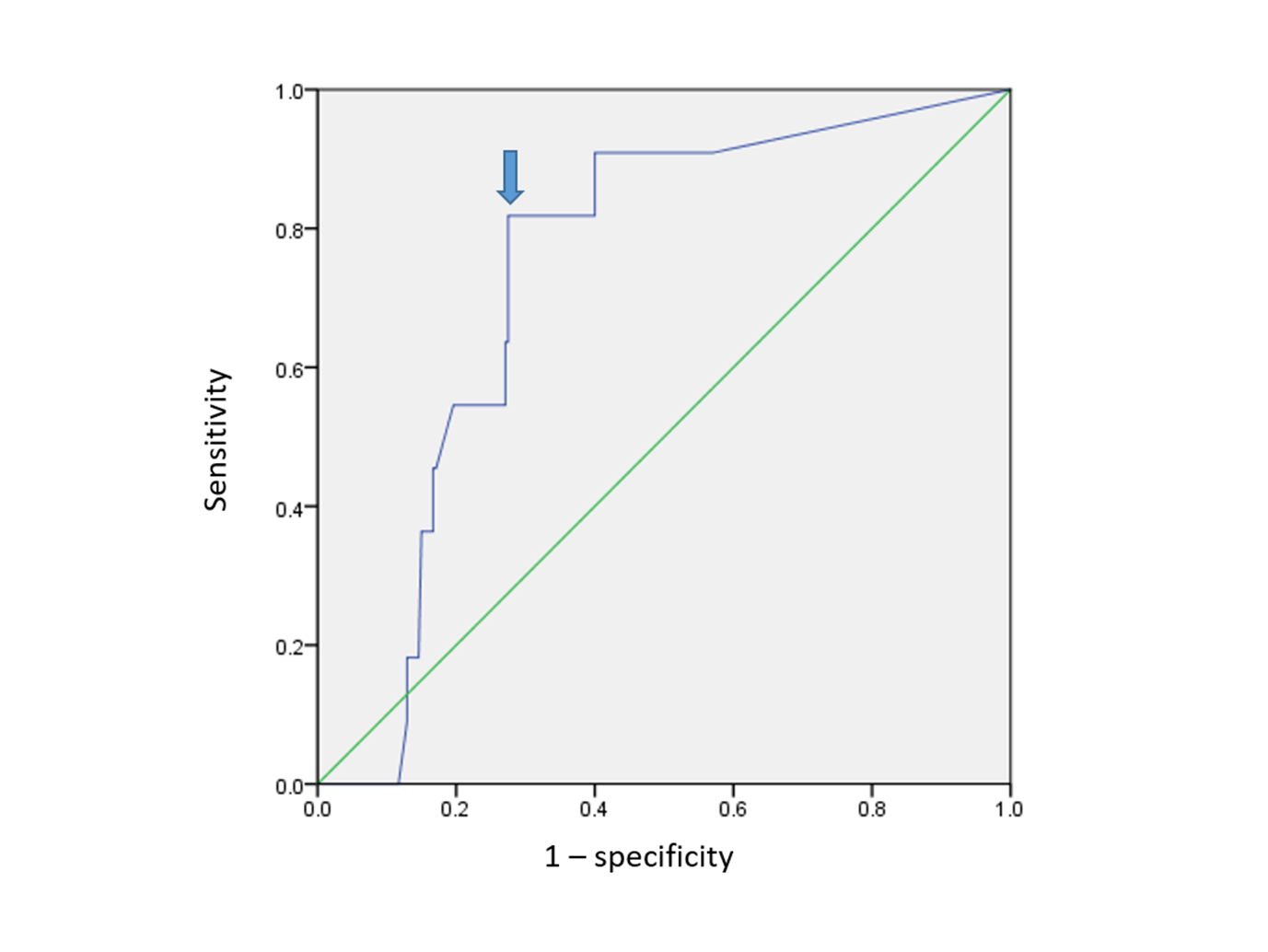


**Supplementary figure S1**. ROC curve for determining the optimal cutoff value of the lymph node ratio
